# Gut mycobiome dysbiosis in rats showing retinal changes indicative of diabetic retinopathy

**DOI:** 10.1371/journal.pone.0267080

**Published:** 2022-04-19

**Authors:** Shalem Raj Padakandla, Taraprasad Das, Gumpili Sai Prashanthi, Kiran Kumar Angadi, S. Sreenivasa Reddy, G. Bhanuprakash Reddy, Sisinthy Shivaji

**Affiliations:** 1 Prof. Brien Holden Eye Research Centre, L. V. Prasad Eye Institute, Kallam Anji Reddy Campus, Hyderabad, Telangana, India; 2 Smt. Kanuri Santhamma Centre for Vitreo Retinal Diseases, L. V. Prasad Eye Institute, Hyderabad, Telangana, India; 3 Biochemistry Division, ICMR-National Institute of Nutrition, Hyderabad, Telangana, India; University of Hyderabad, INDIA

## Abstract

The current study compared the gut mycobiomes of diabetic rats generated by a streptozotocin chemical challenge, diabetic rats with retinal changes and normal control rats over a period of 4 months. Sustained increase in blood sugar levels (*>*150 mg/dL) confirmed the induction of diabetes. Histology and immunohistochemistry were used to identify changes in the retinal tissues in the diabetic rats indicative of the animals progressing into diabetic retinopathy. Gut mycobiomes generated using faecal DNA, indicated dysbiosis at the genus level in both diabetic (DM) and diabetic rats with retinal changes (DRC) when compared with the control rats. In Tables 3–6 the specific genera that were significantly increased/decreased in DM1 and DM2 and in DRC1 and DRC2 respectively compared to the respective controls CT1-CT4 rats are listed. Further, the mycobiomes of the DM and DRC rats separated into distinct clusters following heat-map analysis of the discriminating genera. In addition, *β*-diversity analysis separated the mycobiomes of DM and DRC rats from that of the control rats, but the mycobiomes of diabetic rats and diabetic rats with retinal changes showed an overlap. Based on the inferred functions of the discriminating genera in the mycobiomes, we speculated that increase in pathogenic fungi might contribute to the inflammatory status both in diabetic rats and rats showing retinal changes.

## Introduction

In Type 2 Diabetes Mellitus (T2DM) patients it has been observed that Diabetic Retinopathy (DR) is a common ophthalmic disorder which could cause blindness [1]. That T2DM is a major factor leading to DR is of great concern since T2DM numbers globally are estimated to increase from 463 million to 700 million in 2045 [2]. It is well established that dysbiosis (alteration in the diversity and abundance at the phyla and genera level) in the gut bacterial microbiome [3–5] and mycobiome [6–8] is associated with people with DM. Recently, we demonstrated dysbiosis in both the gut microbiomes and mycobiomes in people with T2DM and DR compared with the healthy controls [9, 10]. It was suggested that strategies to restore the diversity of the microbiome and mycobiome could reverse the gut dysbiotic changes in people with T2DM and DR. The gut microbiomes and mycobiomes of Streptozotocin (STZ)-induced diabetic mice and rats could be studied to evaluate the underlying mechanisms of DR [11, 12]. A study demonstrated that in diabetic mice (db/db mice) the microbiome, at the genus level, exhibited dysbiotic changes [13]. Simultaneously, the diabetic mice (db/db mice) presented certain features such as activation of retinal microglia, acellular capillaries and infiltration of peripheral immune cells into the retina which are normally seen under conditions of DR [13]. We had compared the gut bacterial microbiome of STZ-induced DM rats and diabetic rats with retinal changes (DRC) with healthy control rats without signs of DM [14] and showed that the gut bacterial microbiome in DRC rats was different from the control rats. In the current study, gut mycobiome differences in DM and DRC rats was assessed and compared with the mycobiomes of control rats. Such studies on mycobiome changes in DM and DR rats have not been reported earlier and open avenues to identify specific fungi associated with T2DM and DRC and help to develop novel therapies for treatment [15, 16].

## Materials and methods

### Animals

Sprague Dawley rats were housed in polypropylene cages at room temperature (22°C ± 2°C), at 50% humidity and exposed to a 12-hour light/dark cycle. Food and water were available *ad libitum*. These animals were reared at the National Institute of Nutrition (Hyderabad, India) and the work was approved by the Institutional Animal Ethics Committee (P9F/IAEC/NIN/5/2018/GBP/SD-98M).

### Induction of DM and DRC in Sprague Dawley rats

Three months old Sprague Dawley rats (48 nos.) with an average body weight of 230 ± 14 g were used to induce DM and DRC as described recently [14]. Briefly, the experiment involved 8 groups of 6 animals each. The four control groups (CT1–CT4) (with 6 animals in each group) were injected with 0.1-M sodium citrate buffer, pH 4.5, which was used as a vehicle [17, 18]. The remaining 24 rats were injected with Streptozotocin (35 mg/kg) intraperitoneally and divided in to four groups DM1, DM2, DRC1 and DRC2 respectively. The rats were sacrificed as per the following schedule: DM1 and DM2 and the corresponding controls CT1 and CT2 rats were sacrificed 1 and 2 months after induction of diabetes; DRC1 and DCR2 and the corresponding controls CT3 and CT4 rats were sacrificed 3 and 4 months respectively after STZ injection. All the above rats were observed for alterations in the blood glucose levels, retinal histology, and fungal microbiomes (mycobiomes) at the end of 1, 2, 3, and 4 months, respectively [14]. Preparation of retinal sections, Haematoxylin and Eosin staining, immunohistochemistry of VEGF and rhodopsin and immunoblotting of Hypoxia-inducible factor-1 Alpha were as reported in our earlier study [14].

### Faecal sample collection, DNA extraction and PCR amplification

Fresh faecal pellets (five or six) were collected from 24 control animals (CT1-CT4, 6 animals in each group), 12 DM rats (DM1-DM2, 6 animals in each group) and 11 DRC rats (DRC1-DRC2, 6 and 5 animals respectively) in a 5 ml cryotube and stored frozen at –80°C. One animal in the DRC2 cohort died during the course of the experiment and faecal samples could not be collected. Genomic DNA was extracted, purified and quantified as reported in our earlier studies [9, 10, 14, 19–21]. ITS2, a region of the fungal ribosomal RNA gene was amplified with the forward and reverse primers ITS3 (5’-GCATCGATGAAGAACGCAGC-3’) and ITS4 (5’-TCCTCCGCTTATTGATATGC-3’) respectively. Sterile nuclease-free water was used for preparing the reaction mix for PCR. PCR was performed using appropriate controls (9, 10, 14).

### Illumina library preparation and amplicon sequencing

Nextera XT Index Kit (Illumina Inc., San Diego, California, USA) was used for preparing the amplicon libraries following the Metagenomic Sequencing Library preparation protocol for ITS [10]. Sequencing of the libraries using Illumina HiSeq 2 X 250 base pair chemistry was outsourced to Xcelris Genomics Pvt. Ltd. (Ahmedabad, India). Sequencing of PCR negative reactions did not yield any fungal reads.

### Taxonomy assignment of sequenced reads

The sequencing reads were assembled as paired-end reads through FLASH [22] and reads with mean Phred score < 25 and chimeric sequences were eliminated with Prinseq-lite [23] and Usearch61 [24] respectively. QIIME pipeline [25] and UNITE OTUs (ITS) version 8.2 [26] were used to cluster the sequences with > 97% sequence similarity for operational taxonomic unit (OTU) picking. Wang Classifier [27, 28] with a bootstrap of 80% was used for taxonomic assignments of *de novo*-OTUs. OTUs containing < 0.001% of the total number of reads were considered as sparse OTUS and excluded from further analysis. ComBat [29] was used for eliminating batch effect in the mycobiomes.

### Diversity analyses of the mycobiomes

R-Vegan 2.4–2 package (http://vegan.r-forge.r-project.org/) was used for generating Rarefaction curves of the mycobiomes and also for determination of alpha diversity (Shannon diversity, Simpson index, number of observed OTUs, and Chao1 index) differences between the mycobiomes. Significant differences in alpha diversity indices between the groups were determined by t-test.

### Identification of differentially abundant taxonomic groups

Differentially abundant taxonomic groups [P < 0.05] in the mycobiomes were determined by Wilcoxon signed rank and Kruskal-Wallis tests. Further, differences at the genera level were determined using non-metric multidimensional scaling (NMDS) plots. The linear discriminant analysis effect size method (https://huttenhower.sph.harvard.edu/galaxy) was also used to observe the mycobiome features significantly associated with DM and DRC at various taxonomic levels.

### Interaction networks between fungal genera in the mycobiomes

Spearman correlation coefficient (r) was applied to determine pair-wise correlations between abundances of different fungal genera and used to generate interaction networks with the help of CoNet [30] in Cytoscape [31].

## Results

Intraperitoneal injection of STZ induced diabetes (DM) in Sprague Dawley rats. Three months after the induction of diabetes, the DM rats showed changes indicative of retinopathy such as decrease in the thickness of retina, increase in the expression of VEGF and HIF-1*α* and decrease in the expression of rhodopsin. These DM rats with retinopathy changes were designated as diabetic retinopathy rats (DRC) [14]. Dysbiotic changes in the gut bacterial microbiome was earlier reported in STZ-induced DM and DRC rats compared to the control rats [14]. Faecal pellets from the same 3 cohorts of animals (CT, DM, and DRC) were used in the current study for gut mycobiome analysis [14].

### Identification of OTUs, rarefaction and alpha diversity analysis

The average HQ sequencing reads in the mycobiomes from the three cohorts were 467,564, 400,240, and 495,416 in CT, DM, and DRC rats respectively. Three samples, DRC1-19, DRC1-20, and DRC1-21 which yielded fewer reads (6383, 6079 and 3100 respectively) were not considered for analysis. From these mycobiomes 361 OTUs (5 reference and 356 *de novo* OTUs) were identified (S1 Table).

**Fig 1:**
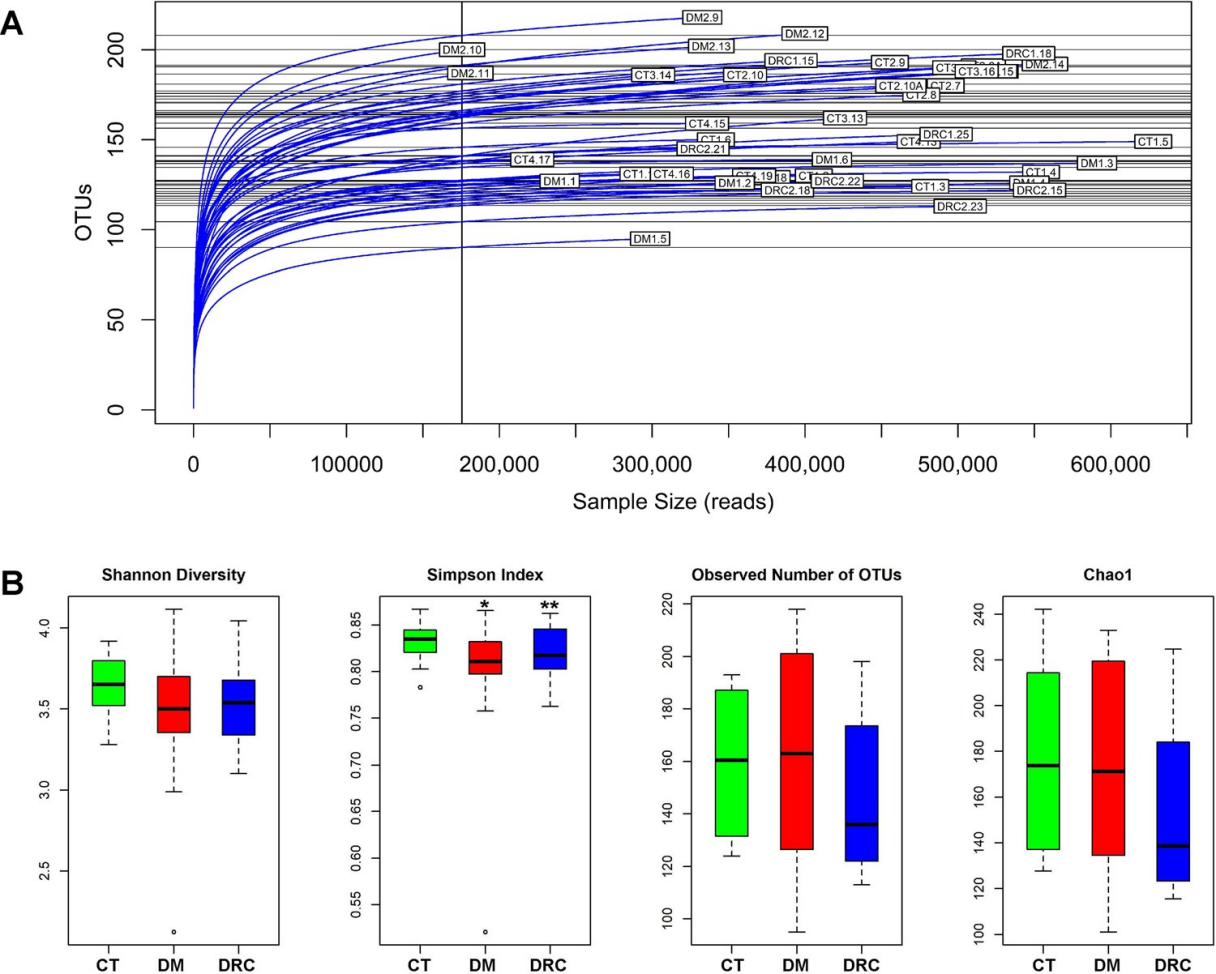
Analyses of the gut mycobiomes of control rats (CT, *n* = 24), diabetic rats (DM, *n* = 12), and diabetic rats showing retinal changes (DRC, *n* = 8) by Rarefaction analysis **(A)** indicating that the depth of sequencing and the sequencing coverage were adequate. In **(B)** the alpha diversity indices (Shannon diversity index, Simpson index, number of observed OTUs, and Chao1 index) showing differences in the mycobiomes of CT, DM and DRC rats. Single asterisk (*) indicates significant differences between the mycobiomes of CT and DM rats and double asterisks (**) between CT and DRC rats as determined by Student’s *t*-test (*P <* 0.05).

The rarefaction curves of the HQ reads of the mycobiomes of the three cohorts showed saturation tendency indicating that the depth of sequencing and the sequencing coverage were adequate and captured the total diversity in the 44 mycobiomes analysed (Fig 1A). Simpson index was the only Alpha diversity index that differed significantly (*P <* 0.05) when the mycobiomes of DM and DRC rats were compared with the control rats; the Shannon index, observed OTUs and the Chao1 index were similar in the mycobiomes of the three cohorts (Fig 1B).

### Analysis of the gut mycobiomes at the phylum level

**Fig 2:**
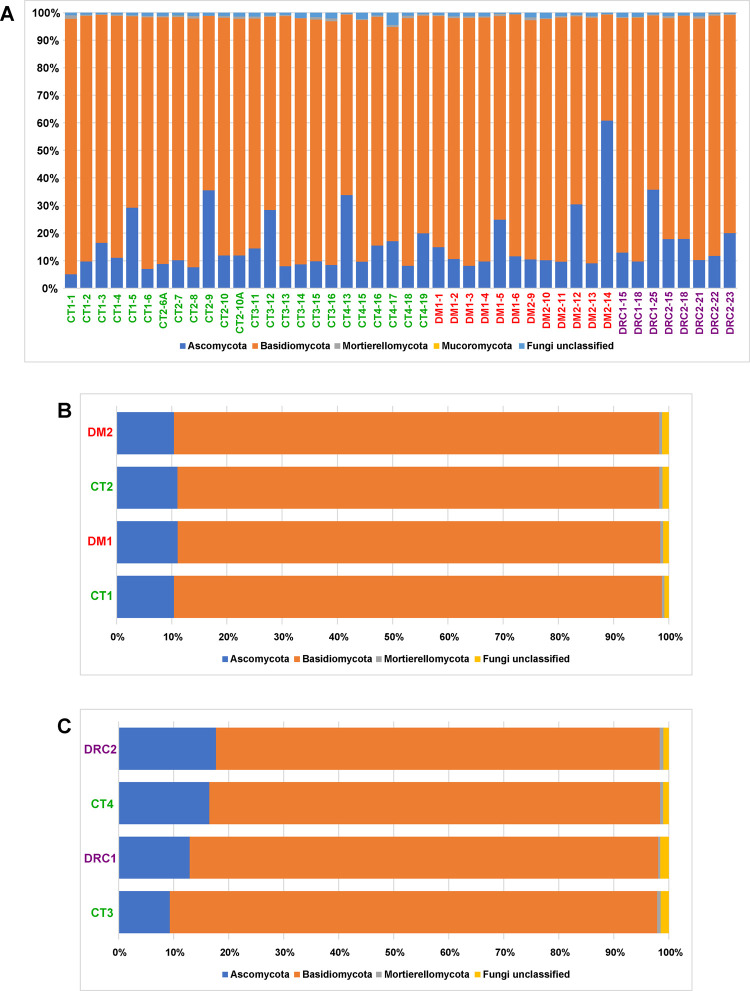
**A.** Changes in the median abundance (%) of fungal phyla in the gut mycobiomes of control (CT1, n = 6; CT2, n = 6; CT3, n = 6; CT4, n = 6), diabetic (DM1, n = 6; DM2, n = 6) and diabetic rats showing retinal changes (DRC1, n = 3; DRC2, n = 5). **B.** changes in the median abundance of fungal phyla from gut mycobiomes of control (CT1, n = 6; CT2, n = 6) and diabetic (DM1, n = 6; DM2, n = 6) rats and **C.** between control (CT3, n = 6; CT4, n = 6) and diabetic rats showing retinal changes (DRC1, n = 3; DRC2, n = 5) rats. DM1 and DM2 are rats after 1 and 2 months of Streptozotocin treatment whereas DRC1 and DRC2 are rats after 1 and 2 months of detection of retinal changes. CT1, CT2, CT3 and CT4 are the corresponding control rats for the DM and DRC cohorts.

Sequences identifying with the phyla Ascomycota and Basidiomycota were consistently detected in CT, DM and DRC mycobiomes. The abundance of Basidiomycota was the highest followed by Ascomycota, Mortierellomycota and Mucoromycota (Tables 1 and 2, Fig 2A–2C). The abundance of the above four phyla in DM and DR were not significantly different from the abundance in CT (P > 0.05).

**Table 1:** Comparison of the median abundance (%) of fungal phyla in the gut mycobiomes of diabetic rats after 1 (DM1, n = 6) and 2 months (DM2, n = 6) with control rats after 1 (CT1, n = 6) and 2 months (CT2, n = 6) respectively.

Phyla	CT1			CT2			DM1			DM2			p value	
	Median	Range	Present out of 6 samples	Median	Range	Present out of 6 samples	Median	Range	Present out of 6 samples	Median	Range	Present out of 6 samples	CT1 vs DM1	CT2 vs DM2
Ascomycota	10.43	5.08–29.21	6	11.07	7.66–35.51	6	11.14	8.18–24.87	6	10.36	9.09–60.82	6	0.82	0.82
Basidiomycota	88.44	69.53–92.75	6	87.21	63.37–90.22	6	87.58	73.89–89.97	6	87.14	38.51–89.13	6	0.59	0.7
Mortierello-mycota	0.47	0.17–1.25	6	0.6	0.08–0.9	6	0.53	0.06–0.86	6	0.52	0.21–1.03	6	0.59	0.7
Mucoromycota	0	0–0	6	0	0–0	3	0	0–0	0	0	0–0.02	3	0.7	0.49
Fungi unclassified	0.8	0.56–1.13	6	1.15	1.04–1.35	6	1.03	0.38–1.3	6	1.22	0.45–1.95	6	0.003	0.61

*DM rats were monitored after 1 (DM1) and 2 (DM2) months respectively after intraperitoneal injection of STZ for the induction of DM.

**Table 2:** Comparison of the median abundance (%) of fungal phyla in the gut mycobiomes of diabetic rats showing retinal changes after 1 (DRC1, n = 3) and 2 months (DRC2, n = 5) with control rats after 3 (CT3, n = 6) and 4 months (CT4, n = 6) respectively.

Phyla	CT3			CT4			DRC1			DRC2			p value	
	Median	Range	Present out of 6 samples	Median	Range	Present out of 6 samples	Median	Range	Present out of 3 samples		Range	Present out of 5 samples	CT3 vs DRC1	CT2 vs DRC2
Ascomycota	9.26	8.01–28.38	6	16.29	8.19–33.79	6	12.95	9.75–35.78	3	17.79	10.32–19.96	5	0.38	0.79
Basidiomycota	88.06	70.2–90.76	6	80.95	65.51–89.86	6	85.14	63.21–88.41	3	81.03	79.18–87.57	5	0.38	0.79
Mortierello-mycota	0.61	0.1–1.04	6	0.54	0.31–0.89	6	0.37	0.29–0.43	3	0.66	0.08–0.83	5	0.55	0.66
Mucoromycota	0	0–0.02	2	0	0–0	6	0	0–0	2	0	0–0	0	0.26	0.93
Fungi unclassified	1.48	0.73–2.04	6	1.03	0.39–4.48	6	1.55	0.58–1.55	3	1.01	0.37–1.29	5	0.48	0.005

*DRC rats were monitored after 3 (DRC1) and 4 months (DRC2) respectively after the induction of DM using STZ.

### Differentially abundant fungal genera in the gut fungal mycobiomes

**Fig 3:**
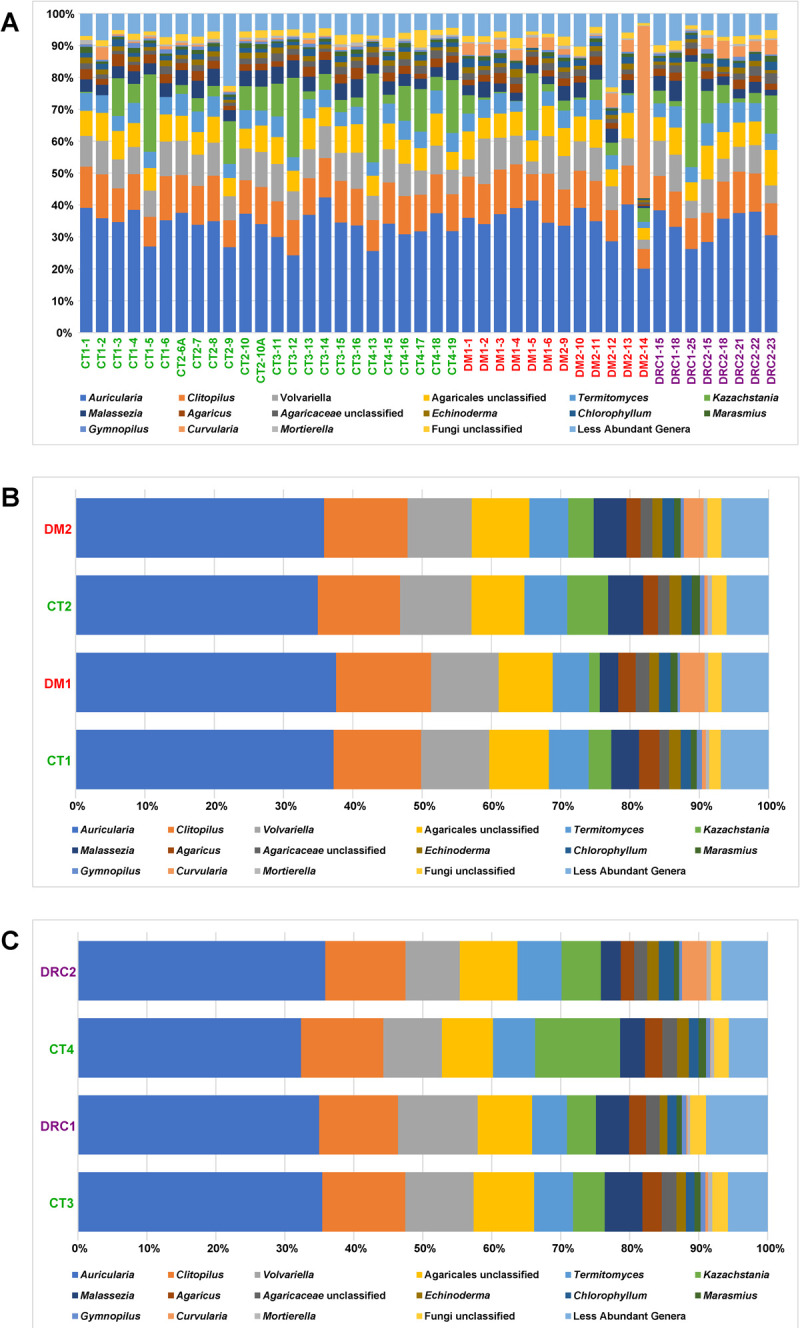
**A.** Changes in the abundance (%) of genera in the gut mycobiomes of control (CT1, n = 6; CT2, n = 6; CT3, n = 6; CT4, n = 6), diabetic (DM1, n = 6; DM2, n = 6) and diabetic rats showing retinal changes (DRC1, n = 3; DRC2 n = 5). **B.** Comparison of median abundance (%) of fungal genera from gut mycobiomes of control (CT1, n = 6; CT2, n = 6) and diabetic (DM1, n = 6; DM2, n = 6) rats and **C.** between control (CT3, n = 6; CT4, n = 6) and diabetic rats showing retinal changes (DRC1, n = 3; DRC2 n = 5).

In the 44 gut mycobiomes of CT, DM and DRC rats 127 genera were identified (S2 Table). The diversity in the gut mycobiomes of the cohorts was not identical (Fig 3A–3C) and did exhibit similarities. CT mycobiomes shared 107 and 97 genera with DM and DRC mycobiomes respectively; CT had 37 unique genera. Further, the mycobiomes of DM and DRC shared 89 genera (S2 Table). Overall, a comparison of the abundance of fungal genera in the mycobiomes of the three cohorts indicated the following:

1. abundance of 3 and 5 genera were significantly decreased in DM1 and DM2 respectively compared to CT1 and CT2 rats (Tables 3 and 4);
2. abundance of 5 and 3 genera were significantly increased in DM1 and DM2 respectively compared to CT1 and CT2 rats (Tables 3 and 4);
3. abundance of 7 and 6 genera were significantly decreased in DRC1 and DRC2 respectively compared to CT3 and CT4 rats (Tables 5 and 6);
4. abundance of 4 and 5 genera were significantly increased in DRC1 and DRC2 respectively compared to CT3 and CT4 rats (Tables 5 and 6).

**Fig 4:**
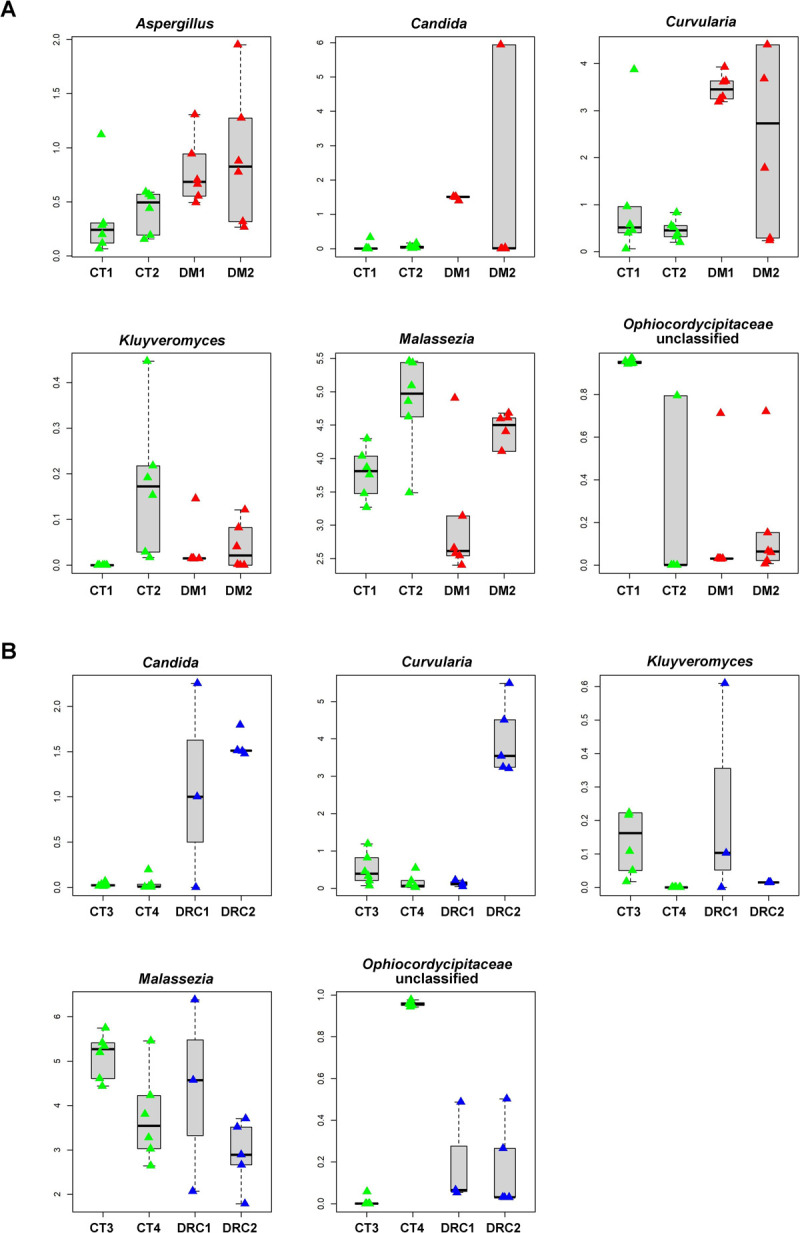
Box plots of genera exhibiting significant (p value < 0.05) differential abundance in gut mycobiomes of control (CT1, n = 6; CT2, n = 6) and diabetic (DM1, n = 6; DM2, n = 6) rats **(A)** and control (CT3, n = 6 and CT4, n = 6), and diabetic rats showing retinal changes (DRC1, n = 3 and DRC2 n = 5) **(B)**. Differentially only genera having a median abundance of > 0.05% in at least one group of samples and significantly different in abundance have been depicted. Median abundances (horizontal line) and inter-quartile ranges are indicated in the plots.

Fig 4 is a comparison of the relative abundance of the discriminating genera in CT, DM and DRC mycobiomes.

**Table 3:** Significant differences in the median abundance of discriminatory genera (>0.01% in any one of the groups; P-value ≤ 0.05) in the gut mycobiomes from control (CT1, n = 6) and diabetic (DM1, n = 6) rats.

S. No.	Genera	Median Abundance (%)		Function
		CT1	DM1	
**Genera decreased in DM1 group**				
1	*Ophiocordycipitaceae* unclassified	0.95	0.03	Plant pathogen
2	*Aureobasidium*	0.01	0	Human pathogen
3	Eurotiales unclassified	0.01	0	Plant / human pathogen
**Genera increased in DM1 group**				
4	*Curvularia*	0.52	3.45	Plant/Human/Animal pathogen
5	*Aspergillus*	0.24	0.68	Human pathogen
6	*Candida*	0	1.51	Human pathogen
7	*Issatchenkia*	0	0.01	Plant/Human pathogen
8	*Kluyveromyces*	0	0.01	Non-pathogenic

**Table 4:** Significant differences in the median abundance of discriminatory genera (>0.01% in any one of the groups; P-value ≤ 0.05) in gut mycobiomes from control (CT2, n = 6) and diabetic (DM2, n = 6) rats.

S. No.	Genera	Median Abundance (%)		Function
		CT2	DM2	
**Genera decreased in DM2 group**				
1	*Trichoderma*	0.38	0.22	Plant pathogen
2	*Alternaria*	0.12	0	Plant/Human pathogen
3	Capnodiales unclassified	0.01	0.01	Plant/human pathogen
4	*Hypoxylon*	0.01	0	Plant pathogen
5	*Pyrenochaetopsis*	0.01	0	Plant pathogen
**Genera increased in DM2 group**				
6	*Nigrospora*	0	0.09	Plant/Human pathogen
7	*Cordyceps*	0	0.01	Animal pathogen
8	*Idriella*	0	0.01	Plant pathogen

**Table 5:** Significant differences in the median abundance of discriminatory genera (>0.01% in any one of the groups; P-value ≤ 0.05) in gut mycobiomes from control (CT3, n = 6) and diabetic rats showing retinal changes (DRC1, n = 3).

S. No.	Genera	Median Abundance (%)		Function
		CT3	DRC1	
**Genera decreased in DRC1 group**				
1	*Clitopilus*	11.54	10.82	Produce antibacterial agents
2	*Echinoderma*	1.33	1.06	Mushroom
3	Capnodiales unclassified	0.02	0	Plant/human pathogen
4	*Cylindrocladiella*	0.01	0	Plant pathogen
5	*Hypoxylon*	0.01	0	Plant pathogen
6	*Kodamaea*	0.01	0	Human pathogen
7	*Pyrenochaetopsis*	0.01	0	Plant pathogen, saprophytic, and endophytic species
**Genera increased in DRC1 group**				
8	*Nectriaceae* unclassified	0	0.09	Plant pathogen
9	*Nigrospora*	0	0.08	Plant/Human pathogen
10	*Gymnopus*	0	0.03	Mushroom
11	*Ustilago*	0	0.01	Plant pathogen

**Table 6:** Significant differences in the median abundance of discriminatory genera (>0.01% in any one of the groups; P-value ≤ 0.05) in gut mycobiomes from control (CT4, n = 6) and diabetic rats showing retinal changes (DRC2, n = 5).

S. No.	Genera	Median Abundance (%)		Function
		CT4	DRC2	
**Genera decreased in DRC2 group**				
1	*Ophiocordycipitaceae* unclassified	0.96	0.03	Animal pathogen
2	*Trichoderma*	0.47	0.16	Plant pathogen
3	*Schizophyllum*	0.11	0.01	Plant pathogen
4	*Aureobasidium*	0.01	0	Human pathogen
5	*Trametes*	0.01	0	Plant pathogen
6	*Xenomyrothecium*	0.01	0	Plant pathogen
**Genera increased in DRC2 group**				
7	*Aspergillus*	0.36	0.55	Human pathogen
8	*Curvularia*	0.07	3.54	Plant/Human/Animal Pathogen
9	*Candida*	0.01	1.51	Human pathogen
10	*Issatchenkia*	0	0.01	Plant/Human pathogen
11	*Kluyveromyces*	0	0.01	Non-pathogenic

### Heat Map and NMDS analysis to differentiate gut fungal mycobiomes

**Fig 5:**
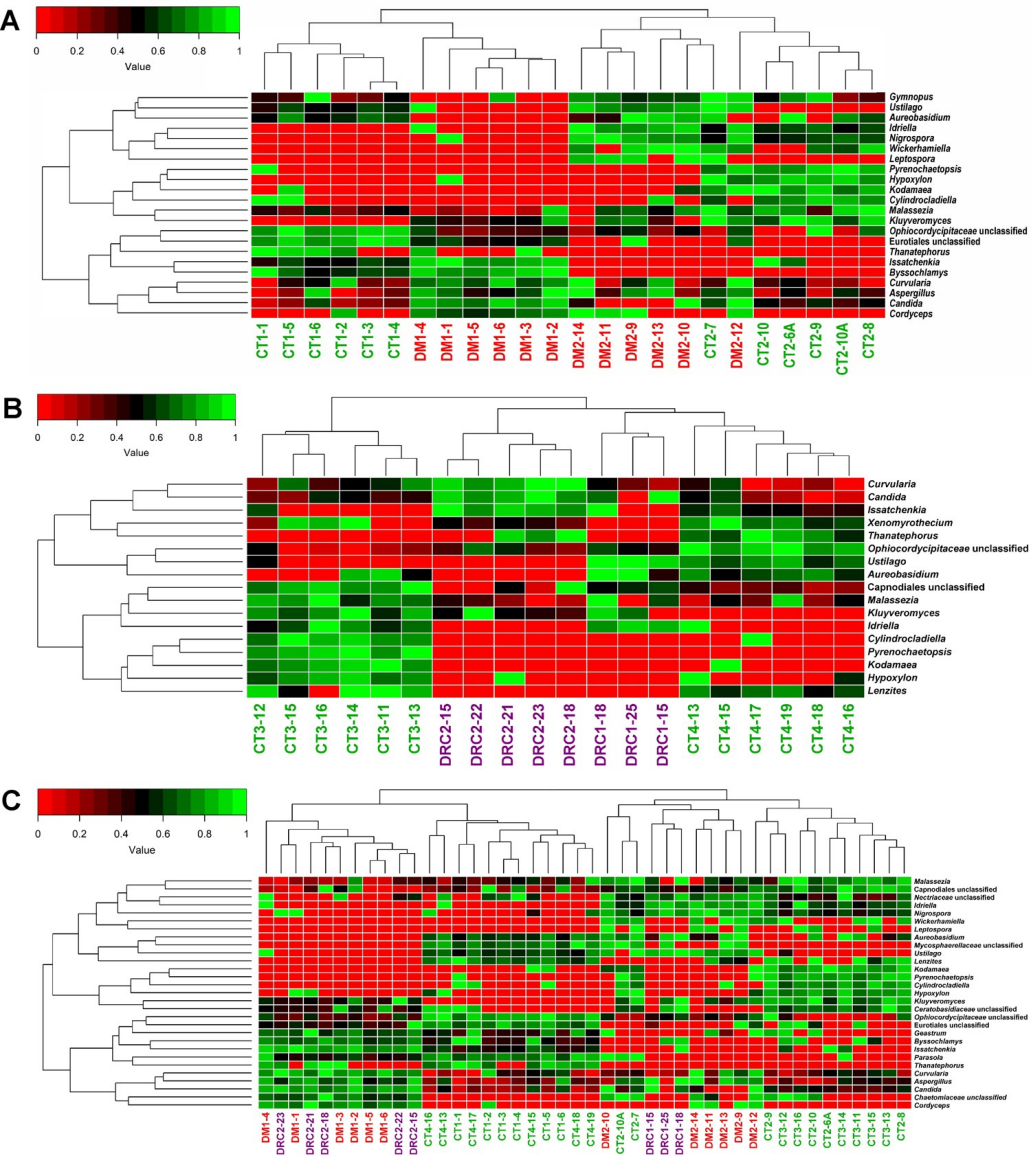
Analyses of the fungal genera (median abundance of >0.01% in at least one group) in the gut mycobiomes by two-dimensional heat map representing rank normalized abundances (scaled between 0 and 1). In **(A)** controls (CT1, n = 6; CT2, n = 6) and diabetic rats (DM1, n = 6; DM2, n = 6), **(B)** controls (CT3, n = 6; CT4, n = 6) and diabetic rats showing retinal changes (DRC1, n = 3; DRC2 n = 5) and **(C)** controls (CT1, n = 6; CT2, n = 6; CT3, n = 6; CT4, n = 6), diabetic (DM1, n = 6; DM2, n = 6) and diabetic rats showing retinal changes (DRC1, n = 3; DRC2, n = 5) are analysed. The discriminating genera were arranged along the two dimensions (axes) based on hierarchical clustering.

Mycobiomes of CT, DM and DRC at the genera level separated into distinct clusters by two-dimensional heat map analysis and the results indicated the following:

- DM1 and DM2 mycobiomes formed separate clades and were affiliated to CT1 and CT2 respectively which also formed two separate clades. The mycobiomes of DM1 and DM2 appeared more closely affiliated (Fig 5A);
- DRC1 and DRC2 mycobiomes also formed separate clades which appeared to be affiliated and were separated from the CT3 and CT4 clades (Fig 5B);
- when the mycobiomes of all the cohorts were analysed together it was observed that CT1 and CT4 grouped together and CT3 and CT2 grouped separately. Distinct clades also represented DM1 and DM2 mycobiomes and DRC1 was affiliated to DM2 whereas DRC2 with DM1 (Fig 5C).

**Fig 6:**
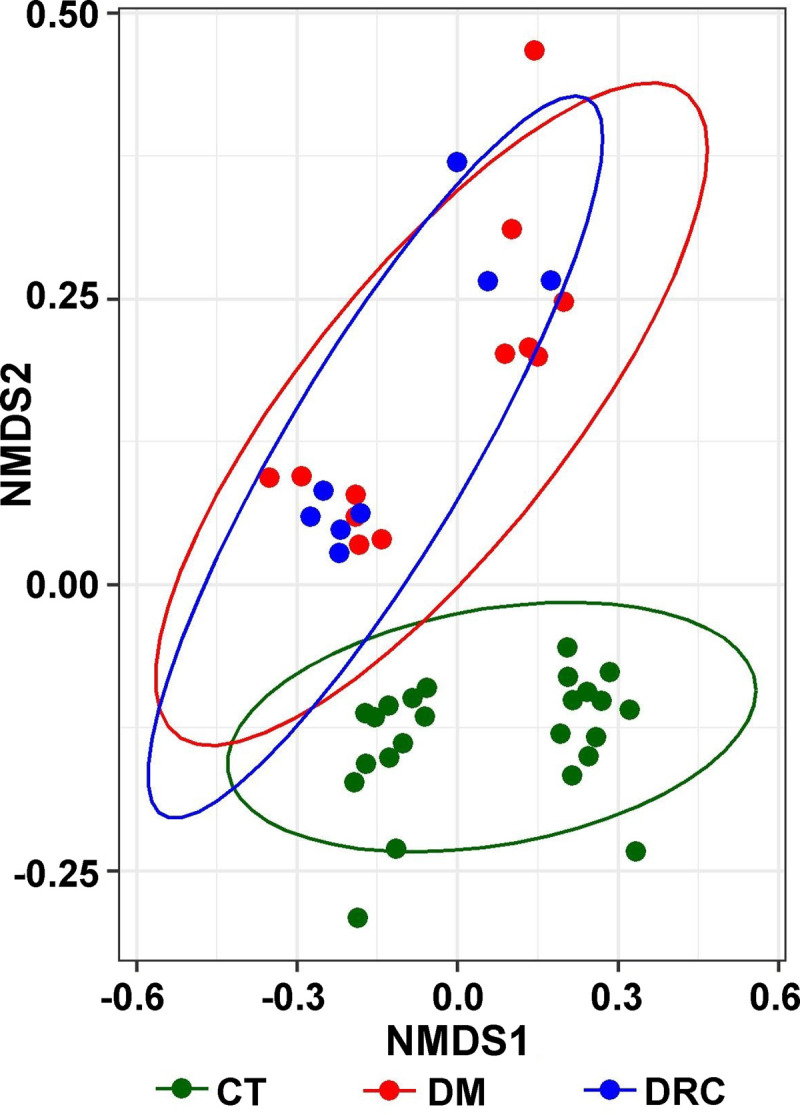
**Two dimensional NMDS plot based on Canberra dissimilarity of mycobiomes at the genera level depicting significant β-diversity differences between control (CT1 –CT4, n = 24, green), diabetic rats (DM 1 and DM2, n = 12, red) and diabetic rats showing retinal changes (DRC1and DRC2 n = 8, blue) (*P* = 0.001).** The gut mycobiomes of CT and DM (*P* = 0.001) and CT and DRC (*P* = 0.001) respectively were significantly different but the mycobiomes of DM and DRC overlapped with each other (*P* = 0.602). The *P*-value was calculated using PERMANOVA.

Beta diversity analysis using NMDS plots based on Canberra dissimilarity also segregated the gut mycobiomes at the genera level of CT and DM (P = 0.001) and CT and DRC (P = 0.001) mycobiomes. But the gut mycobiomes of DM and DRC overlapped with each other (P = 0.602) (Fig 6) (S1 Fig). The P-values were calculated using PERMANOVA.

Mycobiomes of CT, DM and DR at various taxonomic levels are different was also obvious from Linear discriminant analysis effect size method (LEfSE) as indicated by the following colour code: increase in CT-red; increase in DM-green and increase in DRC-blue (S2 Fig).

### Interactions among the genera in the mycobiomes

Interaction networks of mycobiomes (CT, n = 24; DM, n = 12 and DRC, n = 8) using pair-wise correlations between fungal genera abundances in the mycobiomes showed several ‘hub’ genera (exhibiting *>* 10 positive or negative or both interactions) (S3 Fig). Nine hub taxa (*Amarenomyces*, *Kluyveromyces*, *Mycosphaerellaceae* unclassified, *Ophiocordycipitaceae* unclassified, *Orbiliaceae* unclassified, *Pyrenochaetopsis*, *Sympodiomycopsis*, *Thanatephorus* and *Wallemia*) were observed to be unique to the CT group. 6 taxa (*Byssochlamys*, *Ceratobasidiaceae* unclassified, *Humicola*, *Leptospora*, *Polyporaceae* unclassified and *Strelitziana*) were unique to DM and 11 taxa (*Blumeria*, Capnodiales unclassified, *Coniothyrium*, *Cordyceps*, Eurotiales unclassified, *Geastrum*, *Gjaerumia*, *Omphalotus*, *Parasola*, *Pseudozyma* and *Xenomyrothecium*) were unique to DRC. CT and DRC cohorts shared two hub genera, *Iderella* and *Ustilago* and only two hub genera (*Aureobasidium*, *Saccharomyces*) were common between DM and DRC cohorts. The CT, DM and DR interaction networks were different.

## Discussion

Fungi constitute less than 1% of the microbes in the intestinal tract of humans [32] but are critical in maintaining the function of the gut microbiome [33]. Dysbiosis, in the gut mycobiomes is associated with several intestinal disorders including colitis, colorectal cancer, Crohn’s disease, diarrhoea, inflammatory bowel disease etc. [34–41] and other diseases like obesity [42], anorexia nervosa [43], hepatitis B cirrhosis, allergic pulmonary disease, and chronic hepatitis [34, 44]. Earlier studies had also documented dysbiosis in the gut mycobiome [6–8] in people with DM but without any reference to the mycobiome status in DR patients. Recently, we demonstrated that gut mycobiomes are altered both in people with T2DM and DR [10].

### The novelty of the study

Compared with the earlier published studies the current study includes the following novel features:

1. Mycobiome changes were monitored in STZ-induced diabetic rats after the first and second month corresponding with early diabetes without retinal changes and in the third and fourth months after injection of STZ, coinciding with the occurrence of retinal changes [14].
2. Except for our study [14], in none of the earlier studies simultaneous monitoring of changes in retinal markers and gut mycobiome was undertaken.

### Gut mycobiome in the control rats

In accordance with earlier reports in rats, mice, and human beings [10, 45–48] the phyla Basidiomycota and Ascomycota were the predominant phyla. Further, 125 genera were identified in the control rats out of which (S1 Table) *Aspergillus*, *Candida albicans*, *Alternaria*, *Saccharomyces*, *Wallemia*, *Rhodotorula*, *Cladosporium*, unclassified taxon were detected in the rat gut mycobiome as reported earlier by Botschuijver et al. [49]. We did not detect *Monographella*, *Davidiella*, *Vishniacozyma*, *Rhodosporidiobollus*, *uncultured Ascochyta*, *Verticillium*, *Sporobolomyces*, *Cystofilobasidium* in the current study; this difference inability to detect genera that were reported earlier could be attributed to diet differences or the influence of caging and bedding [50]. Several of these genera (*Candida*, *Saccharomyces*, *Aspergillus*, *Cladosporium* and *Alternaria*) identified by us had also been identified in the gut mycobiome of mice [50]. In human beings, 15 most abundant fungal genera were *Saccharomyces*, *Cyberlindnera*, *Malassezia*, *Aspergillus*, *Candida*, *Penicillium*, *Agaricus*, *Cladosporium*, *Fungi spp*., *Fusarium*, *Galactomyces*, *Pichia*, *Alternaria*, *Debaryomyces*, and *Clavispora* [10, 44, 47, 51–55]. Some of the genera like *Muco*r and *Malassezia* were not identified by culture-based analysis implying that they are unlikely inhabitants of the gastro-intestinal tract [47]. These discrepancies in the detection of fungi between individuals are attributed to differences in the sampled population with respect to their geographic origin and diet.

### Gut mycobiome dysbiosis in diabetic rats

This study demonstrated that the phyla Basidiomycota, Ascomycota, Mortierellomycota and Mucoromycota (Table 1, Fig 2A–2C) were not significantly different between CT and DM but distinct differences could be detected between CT and DM, with 8 genera increased in abundance (*Curvularia*, *Aspergillus*, *Candida*, *Issatchenkia*, *Kluyveromyces*, *Nigrospora*, *Cordyceps and Idriella*) (Fig 3A–3C; Tables 3 and 4). All these are plant and human pathogens involved in several disease including DM, except *Kluyveromyces* which is non-pathogenic. *Curvularia* causes orbital cellulitis, rhinosinusitis [56, 57] and onychomycosis [58] in diabetic patients [56, 57]; *Aspergillus flavus* causes otitis [59]; *Candida* causes several skin infections including mucormycosis in people with diabetes [60]; *Nigrospora* produces antidiabetic activity molecules [61]; *Cordyceps militaris* extracts show antidiabetic, hypolipidemic, and even antinephritic effects [62]. Thus, these genera may support the DM status. Concomitantly, the abundance of 8 taxa which are plant/human pathogens decreased in DM1 and DM2 (Tables 3 and 4) and included *Ophiocordyceps sinensis* (*Ophiocordycipitaceae)* which is associated with both diabetes and diabetic nephropathy (DN) [63]; *Aureobasidium*, exhibits antidiabetic effect [64]; *Alternaria* cause ocular infections [65]; the remaining 5 genera (*Eurotiales*, *Trichoderma*, *Capnodiales*, *Hypoxylon* and *Pyrenochaetopsis)* could not be linked to diabetes but are known pathogens. In human beings, studies had indicated that in T2DM patients twenty-one genera decreased in abundance. Most of the genera were plant/human pathogens and also included a few commensal fungi, non-pathogenic fungi and fungi with antimicrobial properties [6–8]. Further, the median abundance of *Candida*, *Kodamaea* and *Meyerozyma* which are pathogens along with *Cladosporium* and *Mortierella*) were increased in abundance in people with T2DM [66]. Such an increase in abundance in pathogenic fungi, might exert a pro-inflammatory response and thus facilitate T2DM which is an inflammatory disease [10]. Dysbiosis (at the diversity, abundance and functional level) in the mycobiomes in DM rats compared to healthy control rats is observed in this study.

### Gut mycobiome dysbiosis in diabetic rats with retinal changes

Gut mycobiomes of diabetic rats with retinal changes is analysed and reported for the first time. At the phyla level no significant differences were observed (Table 2). But at the genera level (Tables 5 and 6; Fig 3A–3C) nine genera were increased in abundance in DRC (both DRC1 and DRC2) compared to healthy controls (CT3 and CT4) and majority of them 8 of 9 genera namely, *Nigrospora*, *Aspergillus*, *Curvularia*, *Candida Issatchenkia*, Nectriaceae, Gymnopus and *Ustilago*, except *Kluyveromyces* [67, 68], were pathogens implying that these pathogenic genera are required for the sustenance of the inflammatory status of DRC rats. It was also observed that 7 (Capnodiales unclassified, *Hypoxylon Pyrenochaetopsis*, Ophiocordycipitaceae unclassified, *Trichoderma*, *Schizophyllum*, *Aureobasidium)* taxa decreased both in DM and DRC rats (compare Tables 3–6). Such a decrease in abundance of several genera (12 of 18) was also reported in both T2DM and DR patients [10]. But, the genera *Clitopilus*, *Echinoderma*, *Cylindrocladiella*, *Kodamea*, *Trametes* and *Xenomyrothecium* were decreased in abundance specifically in DRC rats implying that these genera may have a specific role in DRC. This is very likely, since several of these genera *Cylindrocladiella* [69], *Kodamea* [70], *Trametes* [71], and *Xenomyrothecium* [72], are pathogens and may thus have a pro-inflammatory effect. We also observed decrease in the abundance of *Aspergillus*, *Cladorrhinum*, *Pseudogymnoascus*, and *Diutina*, in T2DM patients which are known animal or human pathogens [10]. But it is unclear, how a decrease in abundance of a pro-inflammatory fungi, *Clitopilus* which produces antibacterial agents [73] and *Echinoderma* [74], a mushroom fungus influences DRC status in rats.

### Alpha diversity, Heatmap and beta diversity analyses

Earlier studies in human beings indicated that Chao1 index and the observed number of OTUs were reduced in mycobiomes in the diseased state in several diseases including DR [10], paediatric Inflammatory Bowel Disease [75], anorexia nervosa [43], obesity [42], and ulcerative Colitis [76] compared to the healthy control mycobiomes. Further, increased richness was increased in patients with Crohn’s disease [37, 40] and hepatitis B [77]. In contrast, we observed that only the Simpson index differed significantly between the controls and DM and DRC cohorts (Fig 1B). Heatmap and beta diversity analysis (Figs 5 and 6) also separated the mycobiomes of CT, DM and DRC rats confirming earlier studies that indicated dysbiotic changes in the gut mycobiomes in people with DM [6–8].

### Relevance of the gut mycobiome changes in DM and DRC rats

It was anticipated that controls would have a preponderance of fungi that are commensals and may also have fungi which would have anti-inflammatory properties whereas, in the diseased state (DM and DRC), there would be an increase in pathogens that could cause inflammation and a concomitant decrease in the abundance of commensal bacteria. Such a clear-cut trend in the mycobiomes of DM and DRC compared to the HC was not obvious. But, overall, an increase or decrease in pathogens was observed in DM and DRC compared to the healthy controls. An Increase in the abundance of pathogenic fungi in DM and DRC is also like that observed in allergic asthma [78]. The only studies available on ocular diseases like uveitis (UVT) [79], bacterial keratitis [19] and fungal keratitis [20] indicated a decrease in abundance of fungi with anti-inflammatory or anti-pathogenic effects [19, 79] in the diseased state. Thus, changes in microbiota (at the taxonomic level) might not be common across all diseases.

### Factors that could influence the gut mycobiomes

This study does not provide insight into the mechanism of how changes in the gut mycobiome influence DRC. But it could be similar to gut microbiome dysbiosis activating uveitis. For example, dysbiosis in the gut microbiome under uveitis, triggers the TH17 cells in the intestine. These TH17 cells are uveitis-relevant cells which cross the intestine, enter circulation and reach the eye to cause uveitis [80, 81]. Another possibility is that dysbiosis may be modulating growth factors like VEGF (vascular endothelial growth factor) which is implicated in retinopathy [82, 83].

## Conclusions

- In this study, for the first time, the gut mycobiomes of CT, DM and DRC rats are discriminated at the genera level.
- This study also showed that the mycobiomes in DM and DRC rats could be differentiated at the genera level.
- The data could help to modulate the mycobiomes of DM and DRC rats to restore the functional attributes of the fungi in the mycobiomes as in control rats.
- Research targeted to unravel the functional attributes of the discriminating fungal genera would help to strengthen the use of fungi as therapeutic agents.

## Limitations

Longitudinal mycobiome studies would help to understand the progression of the disease. Confounding factors like diet, age, gender etc., need to be studied to unravel the mycobiome differences in the diseased state.

## Implication

This study on DM and DRC rats could form the basis for future studies on the role of the gut mycobiome by attempting faecal microbiota transplantation or probiotic therapy.

## Supporting information

S1 Fig Two-dimensional NMDS plot based on Canberra dissimilarity of mycobiomes at the genera level when the three cohorts of CT, DM and DRC were analysed in pairs. The output depicted significant β-diversity differences between **(A)** control (CT1 –CT4, n = 24, green) and diabetic rats (DM 1 and DM2, n = 12, red) (*P* = 0.001) and **(B)** CT and diabetic rats showing retinal changes (DRC1and DRC2 n = 8, blue) (*P* = 0.001). But the gut mycobiomes of **(C)** DM and DRC overlapped with each other (*P* = 0.602). The *P*-value was calculated using PERMANOVA.
(TIF)

S2 Fig Differential taxa of CT, DM and DRC selected by linear discriminant analysis effect size analysis. The taxa between are depicted in a different color as follows: increase in CT-red; increase in DM-green; increase in DRC-blue.
(TIF)

S3 Fig Interaction networks of co-occurrence and co-exclusion at genus level in the gut mycobiomes of **(A)** control rats (CT, n = 24), **(B)** diabetic rats (DM, n = 12) and **(C)** diabetic rats with retinal changes (DRC, n = 8). The degree of interaction is indicated by the size of the nodes in the network. Colour of the edges indicates the positive (green) and negative (red) correlations/interactions.
(TIF)

S1 Table Changes in the median abundance of fungal genera in gut mycobiomes from control (CT), diabetic (DM) and diabetic rats showing retinal changes (DRC). (DOCX)

S2 Table Biom table. (XLSX)
