# Supplementary figures and images for: Gut mycobiome dysbiosis in rats showing retinal changes indicative of diabetic retinopathy
Source: PLoS One. 2022 Apr 19;17(4):e0267080. doi: 10.1371/journal.pone.0267080 (PMC9017887; doi:10.1371/journal.pone.0267080)

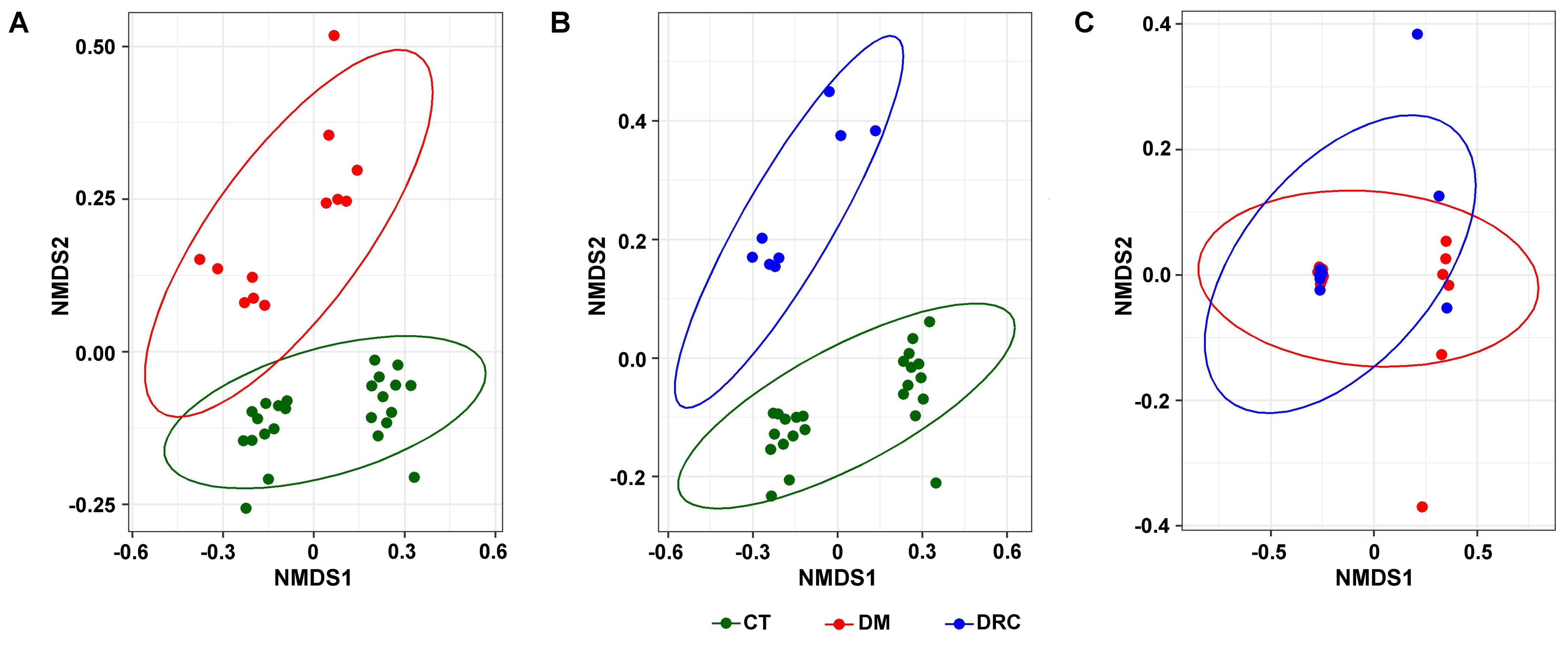

Supplement: S1 Fig — The output depicted significant β-diversity differences between (A) control (CT1 –CT4, n = 24, green) and diabetic rats (DM 1 and DM2, n = 12, red) (P = 0.001) and (B) CT and diabetic rats showing retinal changes (DRC1and DRC2 n = 8, blue) (P = 0.001). But the gut mycobiomes of (C) DM and DRC overlapped with each other (P = 0.602). The P-value was calculated using PERMANOVA. (TIF) [file pone.0267080.s001.tif]

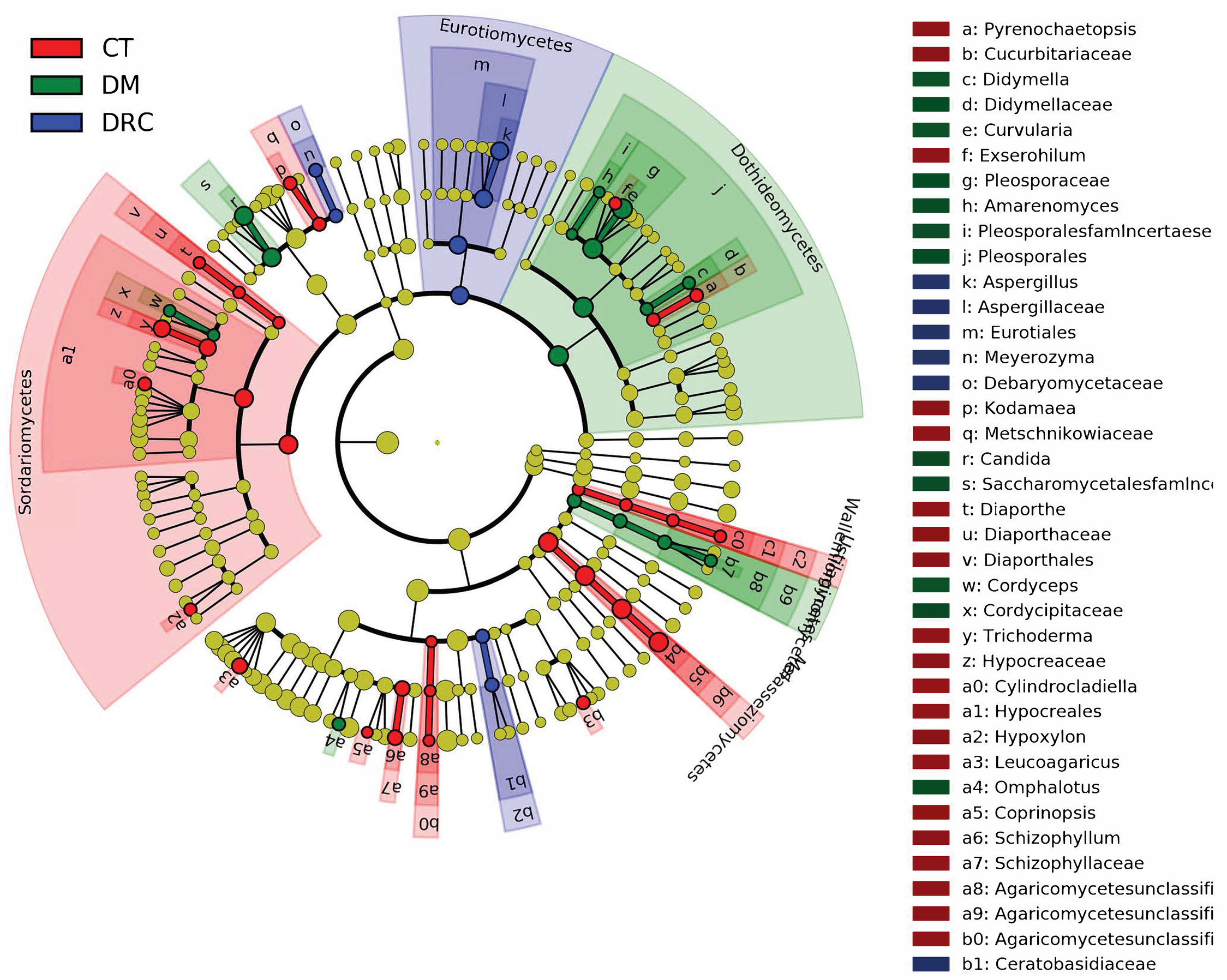

Supplement: S2 Fig — The taxa between are depicted in a different color as follows: increase in CT-red; increase in DM-green; increase in DRC-blue. (TIF) [file pone.0267080.s002.tif]

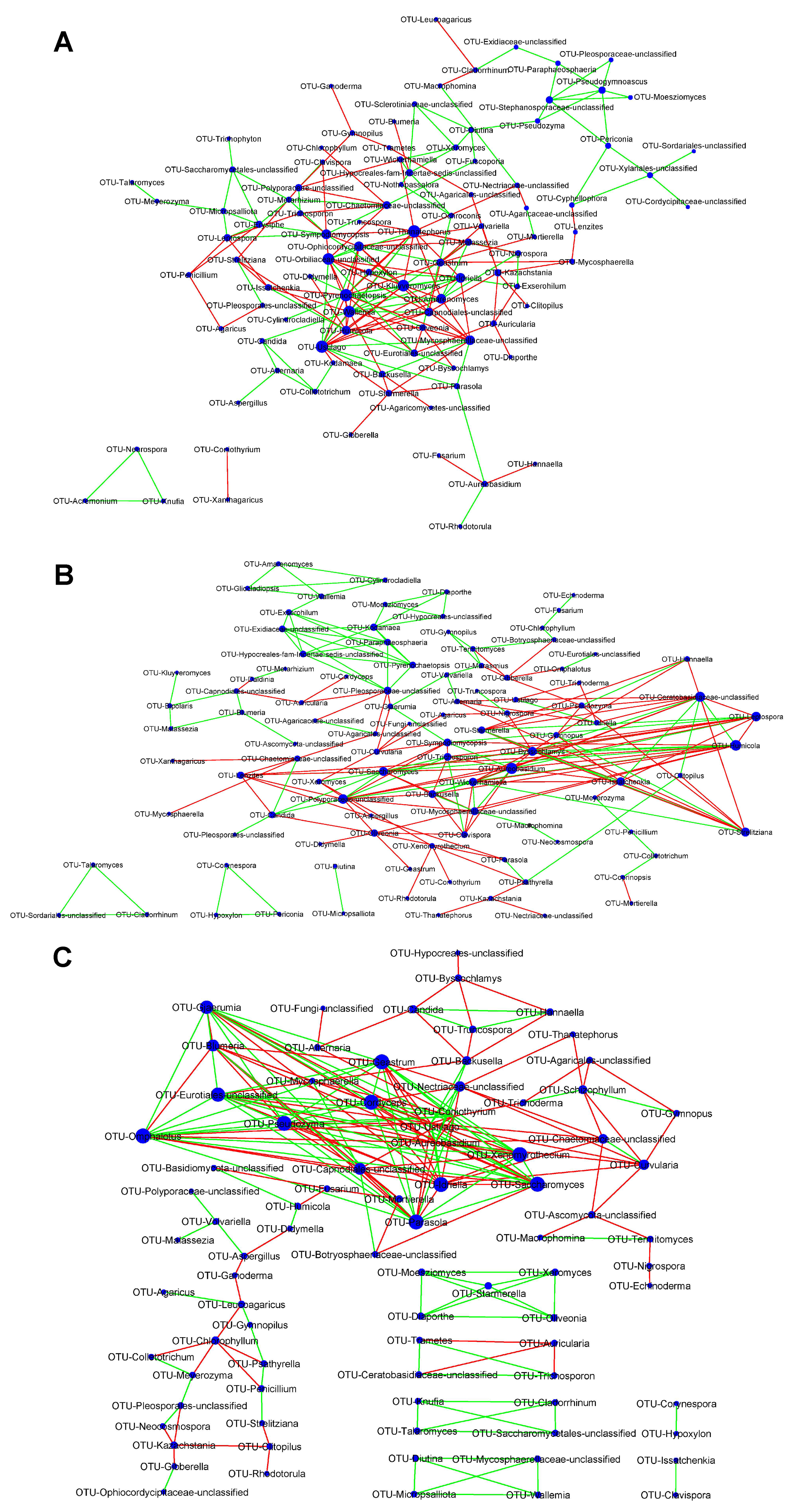

Supplement: S3 Fig — Interaction networks of co-occurrence and co-exclusion at genus level in the gut mycobiomes of (A) control rats (CT, n = 24), (B) diabetic rats (DM, n = 12) and (C) diabetic rats with retinal changes (DRC, n = 8). The degree of interaction is indicated by the size of the nodes in the network. Colour of the edges indicates the positive (green) and negative (red) correlations/interactions. (TIF) [file pone.0267080.s003.tif]
