# Supplementary material for: Gut mycobiome dysbiosis in rats showing retinal changes indicative of diabetic retinopathy
Source: PLoS One. 2022 Apr 19;17(4):e0267080. doi: 10.1371/journal.pone.0267080 (PMC9017887; doi:10.1371/journal.pone.0267080)
Supplement: S1 Table — (DOCX) [file pone.0267080.s004.docx]

**S1 Table. Changes in the median abundance of fungal genera in gut mycobiomes from control (CT), diabetic (DM) and diabetic rats showing retinal changes (DRC).**

| **Genera** | **CT** | | | **DM** | | | **DRC** | | |
| --- | --- | --- | --- | --- | --- | --- | --- | --- | --- |
|  | **Median** | **Range** | **Present out of 24 samples** | **Median** | **Range** | **Present out of 12 samples** | **Median** | **Range** | **Present out of 8 samples** |
| *Acremonium* | 0 | 0-0.07 | 1 | 0 | 0-0 | 0 | 0 | 0-0 | 0 |
| *Agaricaceae unclassified* | 1.78 | 1-2.52 | 24 | 1.79 | 0.6-2.28 | 12 | 1.88 | 0.56-3.25 | 8 |
| *Agaricales unclassified* | 7.9 | 5.6-10.09 | 24 | 7.76 | 3.68-9.51 | 12 | 7.94 | 5.8-11.18 | 8 |
| *Agaricomycetes unclassified* | 0 | 0-0.04 | 22 | 0 | 0-0 | 10 | 0 | 0-0 | 6 |
| *Agaricus* | 2.49 | 1.37-3.78 | 24 | 2.15 | 0.51-3.26 | 12 | 2.2 | 0.86-2.98 | 8 |
| *Alternaria* | 0.05 | 0-0.99 | 16 | 0.01 | 0-0.46 | 9 | 0.02 | 0.01-0.22 | 8 |
| *Amarenomyces* | 0 | 0-0.01 | 13 | 0 | 0-0.04 | 1 | 0 | 0-0 | 0 |
| *Ascomycota unclassified* | 0.06 | 0-0.53 | 24 | 0.04 | 0.03-0.25 | 12 | 0.04 | 0.03-0.16 | 8 |
| *Aspergillus* | 0.34 | 0.07-1.12 | 24 | 0.74 | 0.27-1.95 | 12 | 0.82 | 0.12-3.54 | 8 |
| *Aureobasidium* | 0.01 | 0-0.06 | 19 | 0 | 0-0.11 | 5 | 0 | 0-0.05 | 3 |
| *Auricularia* | 34.34 | 24.26-42.37 | 24 | 35.49 | 20.08-41.43 | 12 | 34.46 | 26.21-38.34 | 8 |
| *Backusella* | 0 | 0-0.02 | 17 | 0 | 0-0.02 | 3 | 0 | 0-0 | 2 |
| *Basidiomycota unclassified* | 0 | 0-0.15 | 24 | 0 | 0-0.07 | 10 | 0 | 0-0.08 | 7 |
| *Bipolaris* | 0.05 | 0-0.43 | 21 | 0.02 | 0-0.31 | 11 | 0.03 | 0.02-0.18 | 8 |
| *Blumeria* | 0.2 | 0.1-0.6 | 24 | 0.23 | 0.06-0.49 | 12 | 0.17 | 0.1-0.58 | 8 |
| *Botryosphaeriaceae unclassified* | 0.4 | 0.06-1.36 | 24 | 0.36 | 0-0.59 | 11 | 0.18 | 0.04-1.33 | 8 |
| *Byssochlamys* | 0 | 0-0.13 | 16 | 0 | 0-0.01 | 6 | 0.01 | 0-0.07 | 7 |
| *Candida* | 0.01 | 0-0.33 | 24 | 1.5 | 0-17.87 | 9 | 1.51 | 0-2.26 | 7 |
| *Capnodiales unclassified* | 0.01 | 0-0.3 | 24 | 0 | 0-0.02 | 9 | 0 | 0-0.07 | 7 |
| *Ceratobasidiaceae unclassified* | 0 | 0-0.05 | 16 | 0 | 0-0.02 | 6 | 0 | 0-0.02 | 7 |
| *Chaetomiaceae unclassified* | 0 | 0-0.08 | 6 | 0 | 0-0.05 | 10 | 0 | 0-0.07 | 5 |
| **Genera** | **CT** | | | **DM** | | | **DRC** | | |
|  | **Median** | **Range** | **Present out of 24 samples** | **Median** | **Range** | **Present out of 12 samples** | **Median** | **Range** | **Present out of 8 samples** |
| *Chlorophyllum* | 1.38 | 0.87-2.24 | 24 | 1.61 | 0.49-2.69 | 12 | 1.54 | 1.1-2.59 | 8 |
| *Cladorrhinum* | 0 | 0-0.15 | 3 | 0 | 0-0.01 | 1 | 0 | 0-0 | 1 |
| *Clavispora* | 0 | 0-0.04 | 17 | 0 | 0-0.03 | 9 | 0 | 0-0.01 | 6 |
| *Clitopilus* | 11.59 | 8.4-14.26 | 24 | 12.35 | 6.16-15.41 | 12 | 10.94 | 9.16-12.99 | 8 |
| *Colletotrichum* | 0.02 | 0-0.2 | 16 | 0 | 0-0.2 | 9 | 0 | 0-0.05 | 4 |
| *Coniothyrium* | 0 | 0-0.1 | 20 | 0 | 0-0.16 | 10 | 0 | 0-0.01 | 5 |
| *Coprinopsis* | 0 | 0-0.01 | 24 | 0 | 0-0.01 | 10 | 0 | 0-0 | 8 |
| *Cordyceps* | 0 | 0-0.01 | 1 | 0 | 0-0.04 | 10 | 0 | 0-0.01 | 5 |
| *Cordycipitaceae unclassified* | 0 | 0-0.06 | 2 | 0 | 0-0 | 0 | 0 | 0-0 | 0 |
| *Corynespora* | 0 | 0-0.07 | 2 | 0 | 0-0.09 | 1 | 0 | 0-0.01 | 1 |
| *Curvularia* | 0.39 | 0.03-3.87 | 24 | 3.45 | 0.24-53.96 | 12 | 3.23 | 0.05-5.49 | 8 |
| *Cylindrocladiella* | 0.01 | 0-0.28 | 15 | 0 | 0-0.09 | 1 | 0 | 0-0 | 0 |
| *Cyphellophora* | 0 | 0-0.05 | 3 | 0 | 0-0 | 0 | 0 | 0-0 | 0 |
| *Daldinia* | 0 | 0-0 | 0 | 0 | 0-0.23 | 2 | 0 | 0-0 | 0 |
| *Diaporthe* | 0 | 0-0.04 | 16 | 0 | 0-0.01 | 2 | 0 | 0-0 | 1 |
| *Didymella* | 0 | 0-0.06 | 5 | 0 | 0-0.1 | 12 | 0 | 0-0.04 | 8 |
| *Diutina* | 0 | 0-0.02 | 1 | 0 | 0-0.02 | 1 | 0 | 0-0 | 1 |
| *Echinoderma* | 1.63 | 0.86-2.37 | 24 | 1.37 | 0.54-2.66 | 12 | 1.13 | 0.83-1.91 | 8 |
| *Erysiphe* | 0 | 0-0.04 | 3 | 0 | 0-0 | 0 | 0 | 0-0 | 0 |
| *Eurotiales unclassified* | 0.01 | 0-0.08 | 16 | 0 | 0-0.03 | 8 | 0 | 0-0.03 | 6 |
| *Exidiaceae unclassified* | 0 | 0-0.1 | 1 | 0 | 0-0 | 1 | 0 | 0-0 | 0 |
| *Exserohilum* | 0 | 0-0.08 | 16 | 0 | 0-0.02 | 2 | 0 | 0-0 | 0 |
| *Fusarium* | 0.21 | 0.05-0.72 | 24 | 0.17 | 0.03-0.61 | 12 | 0.16 | 0.05-0.68 | 8 |
| *Fuscoporia* | 0 | 0-0.04 | 2 | 0 | 0-0 | 0 | 0 | 0-0 | 0 |
| **Genera** | **CT** | | | **DM** | | | **DRC** | | |
|  | **Median** | **Range** | **Present out of 24 samples** | **Median** | **Range** | **Present out of 12 samples** | **Median** | **Range** | **Present out of 8 samples** |
| *Ganoderma* | 0.05 | 0-0.38 | 24 | 0.03 | 0-0.22 | 12 | 0.04 | 0-0.33 | 8 |
| *Geastrum* | 0 | 0-0.03 | 15 | 0 | 0-0.05 | 10 | 0 | 0-0.05 | 5 |
| *Gibberella* | 0 | 0-0.2 | 17 | 0.02 | 0-0.11 | 9 | 0.01 | 0-0.22 | 6 |
| *Gjaerumia* | 0.01 | 0-0.05 | 21 | 0.01 | 0-0.09 | 11 | 0.01 | 0-0.01 | 5 |
| *Gliocladiopsis* | 0 | 0-0.04 | 1 | 0 | 0-0.01 | 1 | 0 | 0-0 | 0 |
| *Gymnopilus* | 0.65 | 0.27-1.59 | 24 | 0.36 | 0-1.05 | 11 | 0.53 | 0.13-1.46 | 8 |
| *Gymnopus* | 0 | 0-0.16 | 24 | 0.01 | 0-0.05 | 7 | 0.01 | 0-0.32 | 6 |
| *Hannaella* | 0 | 0-0.04 | 18 | 0 | 0-0.03 | 7 | 0 | 0-0.03 | 6 |
| *Humicola* | 0 | 0-0.01 | 15 | 0 | 0-0.01 | 7 | 0 | 0-0.01 | 6 |
| *Hypocreales fam Incertae sedis unclassified* | 0 | 0-0.06 | 3 | 0 | 0-0 | 1 | 0 | 0-0 | 0 |
| *Hypocreales unclassified* | 0 | 0-0.04 | 5 | 0 | 0-0.04 | 2 | 0 | 0-0.14 | 2 |
| *Hypoxylon* | 0.01 | 0-0.12 | 14 | 0 | 0-0.06 | 2 | 0 | 0-0.03 | 1 |
| *Idriella* | 0 | 0-0.16 | 13 | 0.01 | 0-0.24 | 7 | 0 | 0-0.03 | 3 |
| *Issatchenkia* | 0 | 0-0.01 | 15 | 0 | 0-0.03 | 6 | 0.01 | 0-0.12 | 6 |
| *Kazachstania* | 5.45 | 0-27.87 | 24 | 2.68 | 0.09-17.88 | 12 | 4.83 | 1.26-33.06 | 8 |
| *Kluyveromyces* | 0.01 | 0-1.54 | 12 | 0.01 | 0-0.15 | 10 | 0.01 | 0-0.9 | 7 |
| *Knufia* | 0 | 0-0.03 | 1 | 0 | 0-0 | 0 | 0 | 0-0.02 | 1 |
| *Kodamaea* | 0.01 | 0-0.3 | 14 | 0 | 0-0.08 | 2 | 0 | 0-0 | 0 |
| *Lenzites* | 0 | 0-0.24 | 19 | 0 | 0-0.01 | 3 | 0 | 0-0 | 0 |
| *Leptospora* | 0 | 0-0.08 | 4 | 0 | 0-0.03 | 4 | 0 | 0-0 | 0 |
| *Leucoagaricus* | 0.2 | 0.01-0.46 | 24 | 0.07 | 0.01-0.56 | 12 | 0.04 | 0.01-0.5 | 8 |
| *Macrophomina* | 0.16 | 0.06-0.44 | 24 | 0.1 | 0.04-0.4 | 12 | 0.19 | 0.07-0.54 | 8 |
| *Malassezia* | 4.37 | 2.64-5.75 | 24 | 3.62 | 0.68-4.91 | 12 | 3.2 | 1.79-6.38 | 8 |
| **Genera** | **CT** | | | **DM** | | | **DRC** | | |
|  | **Median** | **Range** | **Present out of 24 samples** | **Median** | **Range** | **Present out of 12 samples** | **Median** | **Range** | **Present out of 8 samples** |
| *Marasmius* | 0.96 | 0.44-1.95 | 24 | 0.92 | 0.13-1.56 | 12 | 0.74 | 0.26-1.9 | 8 |
| *Metarhizium* | 0 | 0-0.11 | 3 | 0 | 0-0.08 | 1 | 0 | 0-0 | 0 |
| *Meyerozyma* | 0 | 0-0 | 1 | 0 | 0-0.07 | 7 | 0 | 0-0.17 | 4 |
| *Micropsalliota* | 0 | 0-0.04 | 2 | 0 | 0-0.04 | 1 | 0 | 0-0.02 | 1 |
| *Moesziomyces* | 0 | 0-0.01 | 2 | 0 | 0-0.03 | 2 | 0 | 0-0.02 | 1 |
| *Mortierella* | 0.55 | 0.08-1.25 | 24 | 0.52 | 0.06-1.03 | 12 | 0.47 | 0.08-0.83 | 8 |
| *Mycosphaerella* | 0.45 | 0-0.94 | 23 | 0.25 | 0.05-0.8 | 12 | 0.24 | 0.09-0.86 | 8 |
| *Mycosphaerellaceae unclassified* | 0 | 0-0.09 | 14 | 0 | 0-0.08 | 3 | 0 | 0-0.02 | 1 |
| *Nectriaceae unclassified* | 0 | 0-0.25 | 17 | 0.09 | 0-1.32 | 8 | 0.02 | 0-0.2 | 5 |
| *Neocosmospora* | 0.29 | 0.08-0.64 | 24 | 0.26 | 0.03-1.29 | 12 | 0.27 | 0.08-1.18 | 8 |
| *Neurospora* | 0 | 0-0.08 | 2 | 0 | 0-0 | 0 | 0 | 0-0 | 0 |
| *Nigrospora* | 0 | 0-0.11 | 15 | 0.08 | 0-1.38 | 7 | 0.04 | 0-0.37 | 4 |
| *Nothopassalora* | 0 | 0-0.06 | 3 | 0 | 0-0 | 0 | 0 | 0-0 | 0 |
| *Ochroconis* | 0 | 0-0.04 | 2 | 0 | 0-0 | 0 | 0 | 0-0 | 0 |
| *Oliveonia* | 0 | 0-0.06 | 4 | 0 | 0-0 | 3 | 0 | 0-0 | 1 |
| *Omphalotus* | 0 | 0-0.12 | 3 | 0 | 0-0.09 | 8 | 0 | 0-0 | 5 |
| *Ophiocordycipitaceae unclassified* | 0.94 | 0-19.1 | 20 | 0.03 | 0.01-0.72 | 12 | 0.06 | 0.03-0.5 | 8 |
| *Orbiliaceae unclassified* | 0 | 0-0.04 | 13 | 0 | 0-0 | 0 | 0 | 0-0 | 0 |
| *Paraphaeosphaeria* | 0 | 0-0.15 | 2 | 0 | 0-0 | 1 | 0 | 0-0 | 0 |
| *Parasola* | 0 | 0-0.06 | 15 | 0 | 0-0.03 | 7 | 0 | 0-0 | 5 |
| *Penicillium* | 0 | 0-0.37 | 24 | 0.03 | 0-0.17 | 9 | 0.04 | 0-0.23 | 6 |
| *Periconia* | 0 | 0-0.04 | 2 | 0 | 0-0 | 1 | 0 | 0-0 | 0 |
| *Pleosporaceae unclassified* | 0 | 0-0.29 | 2 | 0 | 0-0 | 2 | 0 | 0-0 | 0 |
| *Pleosporales unclassified* | 0 | 0-0.05 | 4 | 0 | 0-0.19 | 3 | 0 | 0-0.14 | 3 |
| **Genera** | **CT** | | | **DM** | | | **DRC** | | |
|  | **Median** | **Range** | **Present out of 24 samples** | **Median** | **Range** | **Present out of 12 samples** | **Median** | **Range** | **Present out of 8 samples** |
| *Polyporaceae unclassified* | 0 | 0-0.04 | 16 | 0 | 0-0.04 | 7 | 0 | 0-0.04 | 7 |
| *Psathyrella* | 0.31 | 0.04-0.94 | 24 | 0.25 | 0.02-0.47 | 12 | 0.2 | 0.03-0.47 | 8 |
| *Pseudogymnoascus* | 0 | 0-0.05 | 1 | 0 | 0-0 | 0 | 0 | 0-0 | 0 |
| *Pseudozyma* | 0 | 0-0 | 2 | 0 | 0-0.07 | 7 | 0 | 0-0 | 5 |
| *Pyrenochaetopsis* | 0.01 | 0-0.26 | 13 | 0 | 0-0.03 | 1 | 0 | 0-0 | 0 |
| *Rhodotorula* | 0.02 | 0-0.15 | 21 | 0.05 | 0-0.11 | 12 | 0.02 | 0.01-0.3 | 8 |
| *Saccharomyces* | 0 | 0-0.03 | 17 | 0 | 0-0.03 | 12 | 0 | 0-0.02 | 8 |
| *Saccharomycetales unclassified* | 0 | 0-0.05 | 1 | 0 | 0-0 | 0 | 0 | 0-0 | 1 |
| *Schizophyllum* | 0.09 | 0-0.4 | 22 | 0.02 | 0-0.11 | 11 | 0.01 | 0.01-0.1 | 8 |
| *Sclerotiniaceae unclassified* | 0 | 0-0.03 | 2 | 0 | 0-0 | 0 | 0 | 0-0 | 0 |
| *Setophoma* | 0 | 0-0.14 | 1 | 0 | 0-0 | 0 | 0 | 0-0 | 0 |
| *Sordariales unclassified* | 0 | 0-0.14 | 2 | 0 | 0-0 | 1 | 0 | 0-0 | 0 |
| *Sordariomycetes unclassified* | 0 | 0-0.15 | 3 | 0 | 0-0 | 0 | 0 | 0-0 | 0 |
| *Spegazzinia* | 0 | 0-0.25 | 1 | 0 | 0-0 | 0 | 0 | 0-0 | 0 |
| *Starmerella* | 0 | 0-0.08 | 5 | 0 | 0-0.01 | 4 | 0 | 0-0.04 | 1 |
| *Stephanosporaceae unclassified* | 0 | 0-0.05 | 1 | 0 | 0-0 | 0 | 0 | 0-0.45 | 1 |
| *Strelitziana* | 0 | 0-0.04 | 4 | 0 | 0-0.01 | 6 | 0 | 0-0.03 | 4 |
| *Subramaniula* | 0 | 0-0.38 | 1 | 0 | 0-0 | 0 | 0 | 0-0 | 0 |
| *Sympodiomycopsis* | 0 | 0-0.06 | 9 | 0 | 0-0.03 | 3 | 0 | 0-0 | 0 |
| *Talaromyces* | 0 | 0-0.1 | 2 | 0 | 0-0.01 | 1 | 0 | 0-0.04 | 1 |
| *Termitomyces* | 5.55 | 4.21-7.23 | 24 | 5.35 | 1.91-6.36 | 12 | 6.09 | 4.33-9.12 | 8 |
| *Thanatephorus* | 0 | 0-0.1 | 10 | 0 | 0-0.07 | 2 | 0 | 0-0.15 | 3 |
| *Trametes* | 0.01 | 0-0.17 | 23 | 0.01 | 0-0.03 | 11 | 0 | 0-0.02 | 8 |
| *Trichoderma* | 0.41 | 0.1-1.51 | 24 | 0.25 | 0.01-0.84 | 12 | 0.18 | 0.08-0.46 | 8 |
| **Genera** | **CT** | | | **DM** | | | **DRC** | | |
|  | **Median** | **Range** | **Present out of 24 samples** | **Median** | **Range** | **Present out of 12 samples** | **Median** | **Range** | **Present out of 8 samples** |
| *Trichophyton* | 0 | 0-0.04 | 2 | 0 | 0-0 | 0 | 0 | 0-0 | 0 |
| *Trichosporon* | 0 | 0-0.18 | 17 | 0 | 0-0.07 | 9 | 0 | 0-0.01 | 7 |
| *Truncospora* | 0 | 0-0.41 | 17 | 0 | 0-0.17 | 11 | 0 | 0-0.01 | 6 |
| *Ustilago* | 0 | 0-0.01 | 14 | 0.01 | 0-0.2 | 7 | 0 | 0-0.02 | 3 |
| *Volvariella* | 9.72 | 7.5-11.66 | 24 | 9.09 | 2.9-14.27 | 12 | 8.35 | 5.48-11.61 | 8 |
| *Wallemia* | 0 | 0-0.11 | 13 | 0 | 0-0 | 2 | 0 | 0-0.02 | 1 |
| *Wickerhamiella* | 0 | 0-0.05 | 7 | 0 | 0-0.05 | 5 | 0 | 0-0 | 0 |
| *Xanthagaricus* | 0.36 | 0.07-0.86 | 24 | 0.32 | 0.07-1.64 | 12 | 0.28 | 0.14-0.48 | 8 |
| *Xenomyrothecium* | 0.01 | 0-0.1 | 19 | 0 | 0-0.03 | 10 | 0 | 0-0 | 5 |
| *Xeromyces* | 0 | 0-0.1 | 3 | 0 | 0-0.04 | 2 | 0 | 0-0 | 1 |
| *Xylariales unclassified* | 0 | 0-0 | 1 | 0 | 0-0.04 | 1 | 0 | 0-0 | 0 |
| *Fungi unclassified* | 1.97 | 1.18-5.46 | 24 | 1.88 | 0.73-3.27 | 12 | 1.83 | 1.11-2.8 | 8 |
